# Blood Pressure, Heart Rate Variability, and Adiposity in Caribbean Pre-pubertal Children

**DOI:** 10.3389/fped.2019.00269

**Published:** 2019-07-10

**Authors:** Morgane Grandemange, Nathalie Costet, Matthieu Doyen, Christine Monfort, Léah Michineau, Marie-Béatrice Saade, Luc Multigner, Sylvaine Cordier, Patrick Pladys, Florence Rouget

**Affiliations:** ^1^CHU Rennes, Endocrinologie-Diabétologie Pédiatrique, Rennes, France; ^2^Univ Rennes, Inserm, EHESP, Irset (Institut de Recherche en Santé, Environnement et Travail) - UMR_S 1085, Rennes, France; ^3^Univ Rennes, Inserm, LTSI - UMR 1099, Rennes, France; ^4^Univ Rennes, CHU Rennes, Inserm, LTSI - UMR 1099, Rennes, France; ^5^Univ Rennes, CHU Rennes, Inserm, EHESP, Irset (Institut de Recherche en Santé, Environnement et Travail) - UMR_S 1085, Rennes, France

**Keywords:** blood pressure, heart rate variability (HRV), adiposity, children, pre pubertal, BMI, fat mass, systolic blood pressure

## Abstract

Childhood obesity prevalence has increased over the last 30 years. The Heart Rate Variability (HRV) studies performed in adults suggest a possible relation between abnormal autonomic regulation and hypertension in the situation of overweight or obesity.

**Objective:** The aims of this study were to explore the early relationships between adiposity and blood pressure and HRV in pre-pubertal children.

**Methods:** Data were collected during the medical examination of the follow-up at 7 years of the TIMOUN mother-child cohort in Guadeloupe. Body Mass Index z-score (zBMI), sum of tricipital and subscapular skinfold thickness, percentage of fat mass, and Waist-to-Height Ratio were measured. A global corpulence score was computed using a Principal Component Analysis (PCA). Systolic Blood Pressure (SBP) and HRV parameters (cardiac holter monitoring) were collected under 2 conditions (calm and tachycardial period). Relations between HRV, SBP, each adiposity indicator and the corpulence score were studied with restricted cubic splines models, and linear regression models. The age at adiposity rebound (AR) was estimated from the individual growth curves.

**Results:** 575 children were included in the SBP study (mean age: 7.7 years, from 85 to 99 months). SBP was linearly correlated with the corpulence score and the zBMI. An increase of 1 in the zBMI was associated with an increase of 2.3 (±0.28) mmHg in SBP. The effect-size of zBMI on SBP was higher in children with early age at AR. Compared to children with normal BMI, children with a zBMI <™2SD had their RMSSD, SDNN, LF and HF indicators in tachycardial conditions significantly reduced by −30, −21, −37, and −48%, respectively. In boys with a zBMI >2SD, we observed a global increase in all HRV parameters (under tachycardial conditions), particularly the LF [β = 0.43 (±0.18)].

**Conclusion:** In pre-pubertal period a positive correlation between adiposity excess and SBP was observed with significant changes of HRV in boys, arguing for an early abnormal autonomic regulation and for early preventive intervention in the infancy period, particularly in case of overweight or obesity. Thinness was associated with a reduction in almost all the HRV parameters studied, when compared to normal corpulence, suggesting a decrease in autonomic influence.

## Introduction

Childhood obesity prevalence has dramatically increased over the last 30 years (1). Childhood obesity is known to be associated with increased cardiovascular risks including hypertension, obesity, and mortality in adulthood (2, 3). Small increase in arterial pressure during young ages can accentuate with time and lead to adult hypertension (4). The trajectory leading to the association of hypertension, abnormal autonomic regulation, and overweight remains poorly established, specifically in the early phases of development including infancy and pre-pubertal period (5). Reduced baroreflex sensitivity and abnormal autonomic regulation measured through Heart Rate Variability (HRV) analyses have been reported in association with both hypertension and obesity. More specifically, abnormal HRV has been observed before and in the early stages of essential hypertension in adults (6) as well as in normotensive obese children (7–9). Taken together, these observations suggest a possible implication of autonomic dysregulation in the developmental origin of hypertension associated with obesity (10, 11).

Our objective was to test the hypothesis of an early association of blood pressure and HRV with corpulence in the pre-pubertal period, in children from the general population.

In the French West Indies, children and women are particularly affected by overweight and obesity. In 2015, the prevalence of obesity in children was higher in Guadeloupe (7.2%) (12) than in metropolitan France (4%) (13). The “TIMOUN” longitudinal mother-child cohort, was set in Guadeloupe to assess the impact of environmental factors on pregnancy, child's health and development and was therefore of particular interest to study the association between cardiac health indicators and corpulence before puberty in children mainly of African ascendance.

## Materials and Methods

### Population

**Figure 1:**
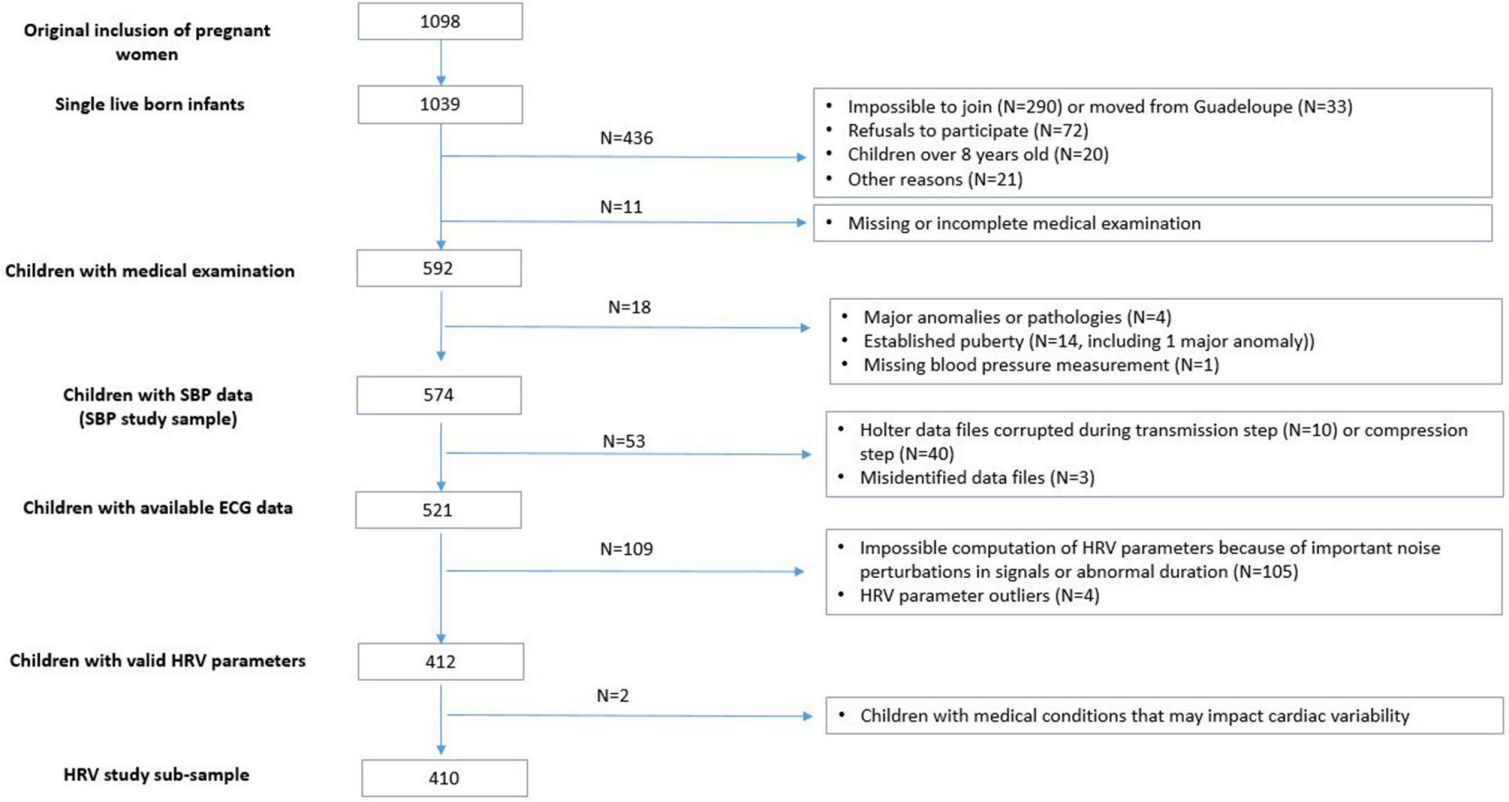
Flow chart of the samples used for analysis.

The TIMOUN study is a prospective epidemiologic mother-child cohort currently underway in Guadeloupe (French West Indies), an archipelago situated in the Caribbean. From December 2004 to December 2007, 1,068 pregnant women were enrolled by obstetricians during their third-trimester check-up visits at public health centers (University Hospital of Pointe-a-Pitre, General Hospital of Basse-Terre, and antenatal care units). Mothers' and newborns' health status was assessed at delivery, and several follow-ups at different ages were conducted afterwards to evaluate the development of the children (14). At 7 years of age, all the children were proposed to participate in a medical examination, with written informed consent obtained from their parents. Among the 1,033 single liveborn infants of the Timoun cohort, 592 children participated in the medical examination (including cardiac holter monitoring) at the university hospital (CHU Pointe-à-Pitre) (detailed flow chart in Figure 1). For our analysis, we excluded 4 children with severe congenital anomaly or disease in childhood (2 children with Down syndrome, 1 with leukemia and 1 with a severe cardiac disease) and 13 children (1 boy and 12 girls) with established puberty (testis volume ≥5 ml for boys and Tanner stage ≥3 for girls). Among the 575 remaining children, 1 had missing blood pressure measurement.

Because of technical or manual errors during the recording or exporting process of the holters data, 521 children had ECG data available (*N* = 53 unavailable). Among them, 105 children had unexploitable ECG signals (important noise perturbations, abnormal duration) and 4 had HRV parameters outliers. Among the 412 children with available and valid HRV parameters, we excluded 2 more children for medical conditions that may impact their cardiac parameters (patent ductus arteriosus and persistent tachycardia). The final sample for the HRV study included 410 children (see flowchart in Figure 1).

### Methods

#### Data Collection

The medical examination lasted about 1 h and was conducted by five trained nurses or midwives, following a standardized protocol. It included physical examination (anthropometric measurements, Tanner stages), cardiac holter, blood pressure measurements, earing test, functional respiratory test, questionnaire, and a blood and urine sample collection at the end. The order in which the different tasks were completed was standardized (Figure S1) and strictly respected for each child. During the medical examination of the child, his/her accompanying parent (most often, the mother) was interviewed with a detailed questionnaire about the sociodemographic characteristics of the family, medical history, dietary habits, life style, and leisure activities of the child. Anthropometric measurements were conducted to assess the child's growth, corpulence and fat repartition and included: height, brachial circumference, waist and hip circumferences, tricipital and subscapular skinfold thickness. Each measurement was assessed twice and a third one was done when the gap between the first two measurements was higher than 0.5 cm (1 mm for skinfolds). Inter-judge assessment sessions were regularly performed between investigators and the pediatrician instructor, in order to guarantee reproducibility of the measurements between investigators and over time. Weight and body fat mass were estimated through bioelectrical impedance analysis, using a professional body composition foot-to-foot monitor (Tanita BC.420.MA-S scale, TOKYO). Pubertal development was assessed using Tanner stages for girls and a Prader orchidometer for testicular volume measurement for boys.

Systolic, diastolic and mean blood pressures were measured twice during the examination, while the child was sitting, at the beginning and at the end of the examination, using an automated blood pressure monitor (Dinamap CARESCAPE V 100, GE Medical Systems Information Technology, Inc. Milwaukee, WI, USA) with an adapted pediatric blood pressure cuff. The first measurement was performed just after a period of standing followed by 3 min sitting (corresponding to the holter monitoring set up), the second measurement was performed in the sitting position, after a long calm period (around 20 min) corresponding to the administration of the questionnaire to the mother. The trained nurses and midwives set up the cardiac holter monitoring at the very beginning of the medical examination and recorded until the end, i.e., during about 1 h (median length = 51 min, Q1 = 44, Q3 = 59), using the SpiderView holter monitor (SORIN, Milan, Italy) with high resolution (1,000 Hz). During recording, children were all submitted to the same examinations or tasks, in the same order (Figure S1).

#### Variables of Interest

##### Adiposity indicators

The body mass index [BMI, calculated as weight (kg)/height (m^2^)], which is the gold standard to define adiposity in childhood (15), was expressed in z-score (z-BMI) according to the Lambda-Mu-Sigma method (WHO) (16) (Supplemental Material). Percentage of Fat Mass was assessed by bioelectrical impedance analysis. The sum of skinfold thickness (sum of the thicknesses of triceps and subscapular skinfolds, in centimeters) and the Waist-to-Height Ratio (WtHR) (cm/cm), which has been proposed for detection of central obesity (17, 18) were also studied.

##### Blood pressure and heart rate variability (HRV) parameters

Systolic Blood Pressure (SBP) was used as the main continuous variable to quantify arterial pressure in children because it has been previously correlated with the occurrence of hypertension in adulthood (4).

HRV was estimated from the holter recorded data using cardiac cycle length time series (RR intervals) and QT duration on two 5 min different periods. These periods corresponded to contrasted situations named as “calm conditions” and “tachycardial conditions”: (i) the most stationary 5 min RR sequence (19) taken from a calm 20 min period (child at rest, quiet, and sitting) and (ii) the most 5-min tachycardial sequence taken from a 10 min-period immediately preceding the blood sample collection (Figure S1). The tachycardial condition was used as an approximation of a stressful condition. The RR time series obtained by using a Multi-Feature Probabilistic QRS Detector^1^ were studied in time domain and frequency domain. The variables studied in time domain were: Mean heart rate, standard deviation of the cardiac cycle length duration (SDNN: SD representing global HRV), and root mean square of the successive difference of RR intervals (RMSSD representing vagally mediated and respiratory related HRV). The frequency domain analysis was performed on RR series resampled at 4 Hz and power spectrum measured using an autoregressive Burg's lattice-based method with an order *p* = 12. The variables studied were obtained from two frequency bands: (i) between 0.04 and 0.15 Hz for the Low Frequency which is not closely correlated with baroreflex gain but is known to be mainly determined by blood pressure ripples (20) (LF); (ii) 0.15–0.4 Hz for the vagally mediated and respiratory related High Frequency (HF); (iv) LF/HF ratio was used as an indicator of sympatho-vagal balance (21).

QT interval length measured between the start of the Q wave and the end of the T wave using a wavelet-based method was presented as QTc using the classical Bazett correction formula.

### Statistical Analyses

The distributions of the SBP and the HRV indicators under tachycardial and calm conditions were checked for normality. SBP and Heart Rate distributions were symmetrical, while SDNN, RMSSD, LF, HF, and LF/HF had highly skewed distributions and needed to be log transformed (log10) before statistical modeling. We kept adiposity indicators in their respective original measurement scale. The relationships between the different adiposity indicators were tested with Pearson correlation coefficients. We combined the four adiposity indicators using a Principal Component Analysis (PCA). The first extracted factor (84.5% of total variance) was considered as a corpulence score reflecting global adiposity.

The relations between HRV, QTc, SBP, and adiposity indicators were studied using restricted cubic splines models in order to identify some potential non-linear associations. We estimated a model for SBP and each cardiac HRV parameter (as the dependent variable) and the adiposity indicators (as the independent spline variables). As the associations of the different adiposity indicators with cardiac parameters presented very similar shapes (no specific associations), we reported associations with the BMI z-score and the corpulence score only. We also run a linear regression model using the categories of BMI z-scores as predictors of the SBP and HRV parameters. The international (WHO) references by sex and age were used to categorize z-BMI in four categories (≤ −2SD,]−2SD; +1SD],]+1SD; +2SD], > 2SD). The “]−2SD; +1SD]” category was used as the reference category.

All the regression models were adjusted for the following potential confounders, chosen a priori: sex of the child, age at measurement, maternal place of birth (French West Indies, other Caribbean Islands [Haiti, Dominica], Europe) and education (<5 years, 5–12 years, >12 years), TV and videogames times, sport time. The mean time spent by the children watching TV and playing video games during a usual week (including Wednesday and the week-end) was separately categorized into 2 categories using the 3rd quartile as threshold (respectively 15 h for TV and 4.5 h for videogames). The sport time per week was categorized into 3 categories (no sport, ≤ 2 h, >2 h), 2 h being the median time among the children practicing sport. We also adjusted for a binary indicator of an obesogenic diet of child (intake of chips, or pizzas/pies, or snacks at least once a day or candies and confectionery several times a day).

As HRV parameters are known to differ according to sex in adulthood (22) we produced analyses stratified by sex to explore potential sex-specific associations between cardiac and adiposity indicators.

We also conducted a sensitivity analysis of the associations between the BMI z-score and the HRV parameters to the additional adjustment for the child's SBP.

Finally, we studied the interaction of the age at adiposity rebound (AR) in the association between adiposity and the SBP. The age at AR was estimated after modeling the individual growth curves of the children from birth to 7 years of age (adapted Jenss-Bayley non-linear mixed model) (23). After testing interaction between the age at AR and SBP, we used quartiles of the age at AR to stratify the analysis of the association between the BMI z-score and SBP.

Analyses were performed with SAS software (version 9.0.1; SAS Institute, Cary, NC).

## Results

### Study Population

Table 1 presents the children's characteristics according to the sex and to the sub-sample of analysis (SBP or HRV analysis). The 575 children included in the SBP study were evaluated at an average age of 7 years and 8 months (min: 85 months, max: 99 months), girls and boys were equally represented. Fourteen percent of the children were born preterm. The mother's place of birth was predominantly French West Indies (81%). Overweight and obesity concerned, respectively 23 and 18% of the mothers.

**Table 1:** Sample characteristics.

	**Sample for SBP analysis (*****N*** **= 575)**				**Sub-sample for HRV analysis (*****N*** **= 410)**			
**Characteristics**	**Boys (*****N*** **= 283)**		**Girls (*****N*** **= 292)**		**Boys (*****N*** **= 208)**		**Girls (*****N*** **= 202)**	
	***N***	**% or mean (std)**	***N***	**% or mean (std)**	***N***	**% or mean (std)**	***N***	**% or mean (std)**
**MATERNAL CHARACTERISTICS**								
**Maternal place of birth**								
French West Indies	238	84.1	227	77.7	173	83.2	159	78.7
Other Caribbean Islands	25	8.8	27	9.3	19	9.1	16	7.9
Europe	20	7.1	38	13.0	16	7.7	27	13.4
**Maternal education (years)**								
<5	13	4.6	15	5.1	8	3.8	9	4.5
5–12	202	71.4	196	67.1	147	70.7	134	66.3
>12	68	24	81	27.7	53	25.5	59	29.2
**Maternal BMI**								
Underweight	19	6.8	14	4.9	14	6.9	9	4.5
Normal	151	54.3	149	51.9	110	53.9	103	51.8
Overweight	63	22.7	68	23.7	46	16.7	48	19.6
Obese	45	16.2	56	19.5	34	22.6	39	24.1
**CHILD AT BIRTH**								
**Preterm birth**								
Yes	45	15.9	38	13.0	28	13.5	31	15.3
No	238	84.1	254	87.0	180	86.5	171	84.7
**Intra-uterine growth retardation**								
Yes	27	9.5	18	6.2	18	8.6	14	6.9
No	256	90.5	274	93.8	190	91.4	188	93.1
Birth weight	283	3135 (513)	292	3107 (559)	208	3155 (501)	202	3059 (572)
**CHILD AT 7 YEARS**								
Age at examination (months)	283	92.1 (2.7)	292	91.9 (2.6)	208	92.3 (2.4)	202	92.0 (2.6)
**Sport participation**								
No	141	50.2	161	55.0	104	50.0	113	56.0
Yes	140	49.8	131	45.0	104	50.0	89	44.0
Sport time (h/weeks)	140	3.0 (1.6)	131	2.5 (1.7)	104	3.0 (1.7)	89	2.5 (1.7)
Screen time, TV (h/weeks)	283	11.1 (7.2)	292	11.8 (7.7)	208	11.2 (7.5)	202	11.7 (7.3)
**Screen time, video games**								
No	77	27.2	95	32.5	48	23.1	66	32.7
Yes	206	72.8	197	67.5	160	76.9	136	67.3
Video games time (h/weeks)	206	4.9 (4.0)	197	4.4 (4.1)	160	4.8 (3.9)	136	4.3 (4.0)
**Obesogenic food consumption**								
No	233	82.3	255	87.3	171	82.2	173	85.6
Yes	50	17.7	37	12.7	37	17.8	29	14.4

At 7 years of age, 47% of the boys and 41% of the girls practiced sport outside the school. Respectively, 77 and 67% played videogames (with an average duration of 4–5 h per week) and 18 and 14% had obesogenic food habits. They usually watched TV during 11–12 h per week (Table 1).

Table S1 describes adiposity and cardiac indicators measured at 7 years of age in children with SBP measurements available. Table 2 describes the sub-sample of children with HRV data also available. Both samples are very similar.

**Table 2:** Adiposity and cardiac characteristics of the children included in the HRV analysis (*N* = 410).

	**Boys (*****N*** **= 208)**		**Girls (*****N*** **= 202)**	
**Characteristics**	***N***	**% or mean (std) or median (IQR)^**a**^**	***N***	**% or mean (std) or median (IQR)^**a**^**
**ADIPOSITY MEASUREMENTS**				
Weight (kg)	207	28.0 (5.8)	202	28.0 (5.7)
Height (cm)	208	130.6 (5.9)	202	130.2 (5.9)
BMI (kg/m^2^)	207	16.3 (2.5)	202	16.4 (2.4)
BMI z-score	207	0.2 (1.4)	202	0.3 (1.2)
BMI z-score categories				
Underweight, ≤−2SD	10	4.8	3	1.5
Normal, ]−2DS; +1DS]	147	70.7	145	71.8
Overweight, ]+1DS; +2DS]	26	12.5	45	22.3
Obese, >2SD	25	12.0	9	4.5
% Fat mass	208	18.0 (5.5)	202	20.5 (6.3)
Sum of triceps + subscapular skinfolds (mm)	208	14.5 (5.7)	202	18.1 (6.4)
Waist-to-height ratio	208	0.44 (0.04)	202	0.44 (0.04)
**CARDIAC MEASUREMENTS**				
SBP (mmHg)	207	99 (12)	202	98 (13)
**HRV under calm conditions**				
Heart rate (bpm)	208	82.7 (15.3)	202	88.0 (13.6)
SDNN (ms)	208	76.3 (45.2)	202	69.4 (35.7)
RMSSD (ms)	208	77.6 (59.5)	202	68.0 (51.2)
LF (10^3^.ms^2^)	208	0.10 (0.12)	202	0.09 (0.09)
HF (10^3^.ms^2^)	208	0.15 (0.23)	202	0.12 (0.20)
LF/HF	208	0.65 (0.51)	202	0.70 (0.64)
Corrected QT interval (ms)	208	417.4 (25.0)	202	420.1 (24.6)
**HRV under tachycardial conditions**				
Heart rate (bpm)	208	88.0 (13,15)^*^	202	95.3 (14.5)^*^
SDNN (ms)	208	89.0 (46,2)^*^	202	78.8 (36.5)^*^
RMSSD (ms)	208	71.0 (59,7)^*^	202	57.3 (42.7)^*^
LF (10^3^.ms^2^)	208	0.10 (0.12)	202	0.07 (0.08)
HF (10^3^.ms^2^)	208	0.13 (0.25)^*^	202	0.09 (0.14)^*^
LF/HF	208	0.71 (0,57)^*^	202	0.79 (0.56)^*^
Corrected QT interval (ms)	208	424.6 (25.6)^*^	202	427.2 (22.0)^*^

a *Median values are presented for the HRV parameters (except Heart Rate) because of their skewed distributions*.

* *Indicates that measurement differs significantly in tachycardial vs. in calm conditions (paired t-tests on raw or log-transformed data, p < 0.001)*.

The respective repartition of BMI categories in boys and girls was as follows: 69 and 72% had a normal BMI (]−2DS; +1DS]); 12 and 19% were classified as overweight (]+1DS; +2DS]); 15 and 7% were obese. The proportion of fat mass was 18% in boys and 20% in girls, while the WtHR was 0.44 in both boys and girls. The correlations between the four adiposity indicators were high (>0.80 for all of them, except the sum of skinfolds with a 0.77 correlation with the BMI z-score) (Table 3). Correlations tended to be higher in girls (*r* = 0.93 between the BMI z-score and the % Fat Mass in girls vs. *r* = 0.82 in boys) (Table 3). The first factor of the PCA combined them into a global adiposity indicator to which all the four contributed equally (average contribution = 26%) (Figure S2).

**Table 3:** Pearson correlations between adiposity indicators.

	**BMI z-score**	**% Fat mass**	**Sum of skinfolds^**a**^**	**WtHR^**b**^**
**BOYS**				
**BMI z-score**	1	0.82	0.80	0.86
**% Fat mass**		1	0.81	0.81
**Sum of skinfolds**^**a**^			1	0.82
**WtHR**^**b**^				1
**GIRLS**				
**BMI z-score**	1	0.93	0.80	0.85
**% Fat mass**		1	0.86	0.89
**Sum of skinfolds**^**a**^			1	0.83
**WtHR**^**b**^				1

a *Sum of skinfolds: Sum of triceps + subscapular skinfolds*.

b *WtHR, waist-to-height ratio*.

The mean SBP was similar in boys and girls (*t*-test *p* = 0.42) with no correlation of SBP with age (*r* = 0.01; *p* = 0.72). The height of the child at measurement was positively associated with SBP (*r* = 0.25; *p* < 0.0001). SBP did not significantly differ between children whose mother was born in French West Indies and children whose mother was born in Europe, *p* = 0.10). It was significantly higher in children whose mother was born in other Caribbean Islands (*p* < 0.01).

All the HRV parameters differed significantly between boys and girls, except the LF/HF ratio, both in calm and in tachycardial conditions. Boys had significantly lower Heart Rate and significantly higher HRV parameters. They did not differ across the maternal place of birth or the age at the examination. All HRV parameters differed significantly in calm and tachycardial conditions, except LF. In tachycardial conditions, Heart Rate, SDNN, LF/HF, and corrected QT were significantly higher compared to calm conditions, while RMSSD and HF were lower.

None of the HRV parameters were correlated with SBP in calm or tachycardial conditions, except the Heart Rate measured under tachycardial conditions which was moderately correlated (*r* = 0.10, *p* = 0.03) with SBP.

### Association Between SBP and Adiposity at 7 Years of Age

**Figure 2:**
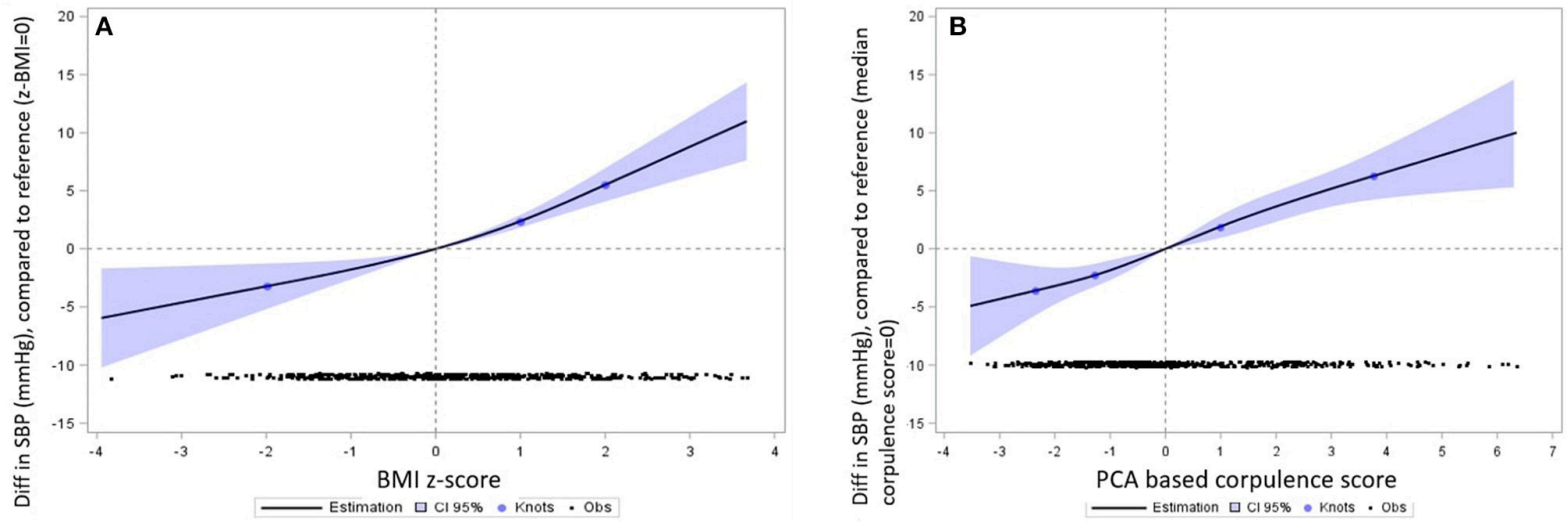
Association between SBP (mmHg) and BMI z-score **(A)** and corpulence score **(B)**. Restricted cubic splines regression models, adjusted for sex of the child, age at measurement, maternal place of birth and education, TV and videogames times, sport time, indicator of obesogenic food consumption. All non-linear associations were non-significant (*p* > 0.05). Interpretation: The reference value for the adiposity indicator (indicated with a vertical dotted line) was 0 for the z-BMI and the median level for the PCA based corpulence indicator. The y-axis represents the mean difference in SBP (mmHg) associated with the adiposity indicator compared to the mean level of SBP associated with the reference level of the adiposity indicator. The black points in the bottom of the graphs represent the number of children observed in the study at each level of the adiposity indicator. For the PCA-based adiposity indicator, the blue points on the spline regression curve represent the knots, set at the 5th, 25th, 50th, and 75th percentiles of the adiposity indicator. For the BMI z-score, the knots correspond to the WHO thresholds defining underweight and overweight and obesity (respectively −2SD, +1SD, +2 SD). The bluish surface around the estimated regression curve represents the 95% confidence interval of the values of variation in SBP estimated from the spline regression model.

**Figure 3:**
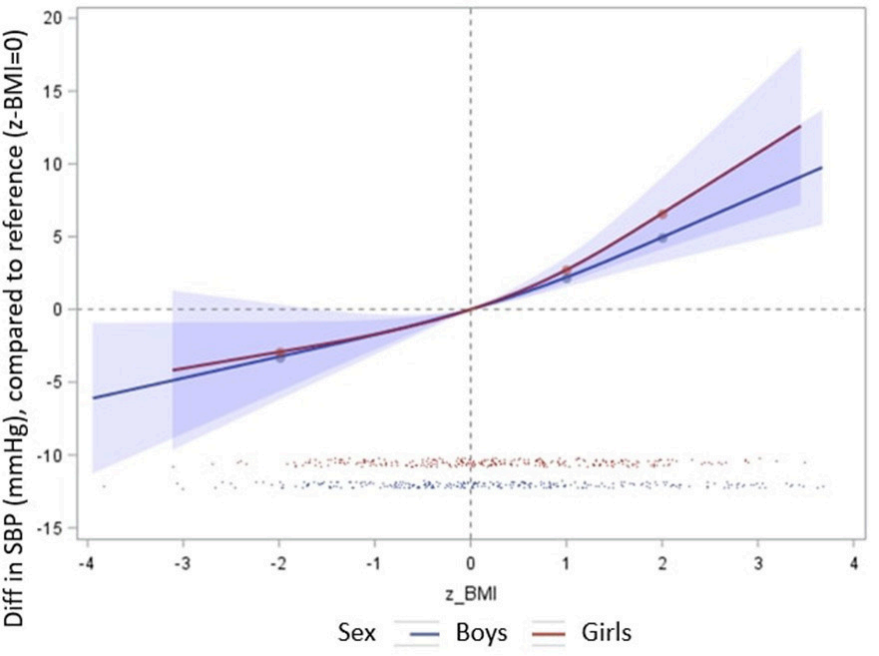
Association between SBP (mmHg) and BMI z-score, according to sex. Restricted cubic splines regression models, adjusted for age at measurement, maternal place of birth and education, TV and videogames times, sport time, indicator of obesogenic food consumption. The y-axis represents the mean difference in SBP (mmHg) associated with BMI z-scores compared to the mean level of SBP associated with a 0 BMI z-score. All non-linear associations were non-significant (*p* > 0.05). Blue line corresponds to boys (1) and red lines to girls (2).

SBP was linearly correlated with the BMI z-score and the corpulence score (Figures 2A,B). This relation was significant from the lowest to the highest values of the adiposity indicators and persisted after adjustment for the confounding factors. In average, an increase of 1 in the BMI z-score (i.e., +1SD in the BMI) was associated with an increase of 2.3 (0.28) mmHg in SBP. No significant sex-specific association was found (sex interaction *p* = 0.3) (Figure 3). The same trend was observed for each of the four adiposity indicators considered independently (data not shown).

As we found a nearly significant interaction between the BMI z-score and the age at the adiposity rebound (*p* = 0.07), we stratified the analyses by the age at the adiposity rebound (AR) (categorical variable). The effect-size of BMI z-score on SBP was decreasing with the age at AR: β = 2.7 (0.9) (*p* < 0.0001) for the children with the earliest AR (first quartile), β = 2.2 (0.52) (*p* < 0.0001) for those with a medium age at AR and β = 0.61 (0.78) (*p* = 0.43) for those with late age at AR (last quartile). The positive association between BMI z-score and SBP was higher in children whose AR has already occurred.

### Association Between HRV and Adiposity at 7 Years of Age

**Figure 4:**
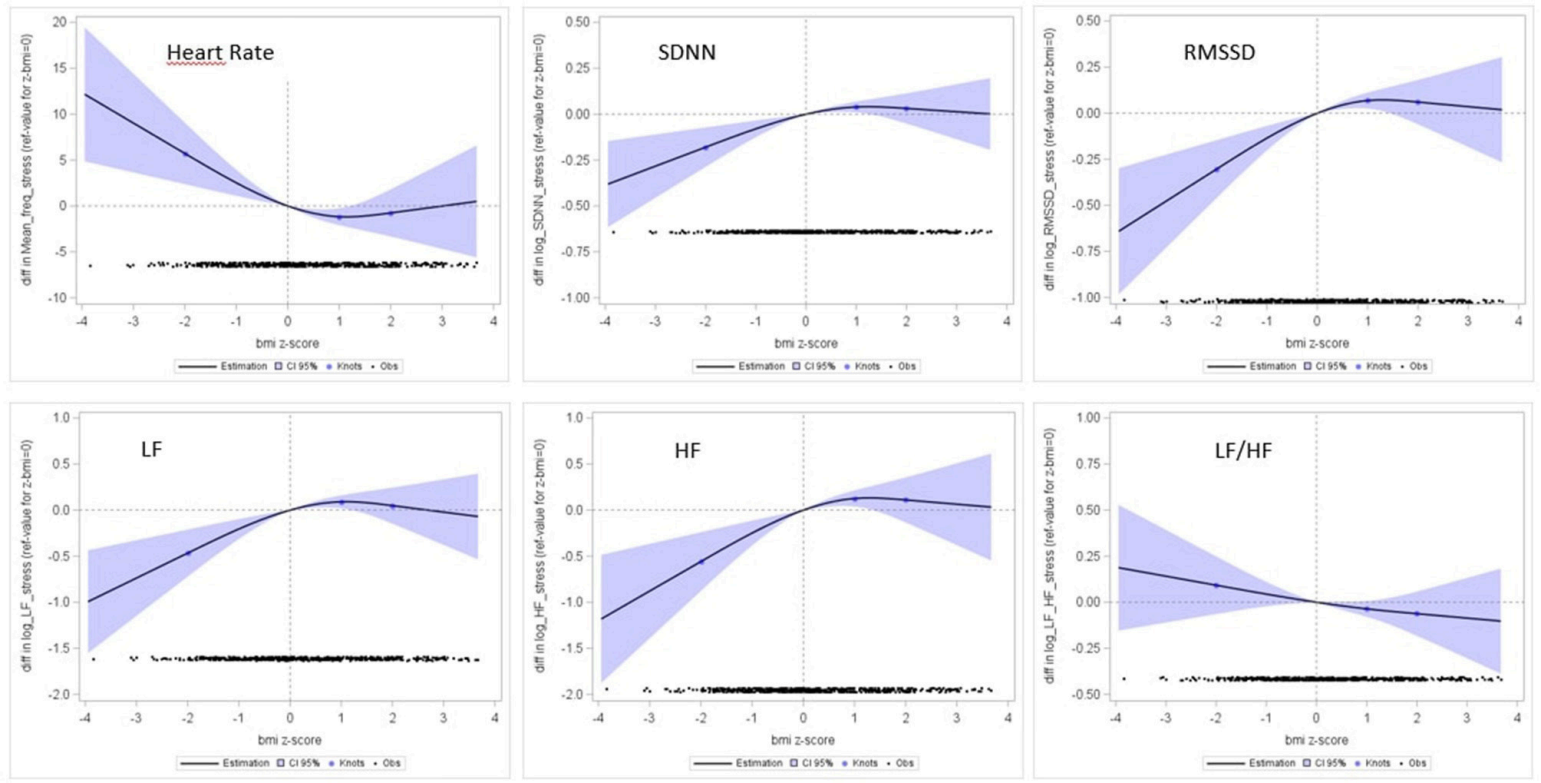
Association between HRV parameters (Y-axis) and BMI z-score (X-axis). Restricted cubic splines regression models, adjusted for age at measurement, maternal place of birth and education, TV and videogames times, sport time, indicator of obesogenic food consumption.

The results of spline regressions of the HRV indicators on the BMI z-score are graphically presented in Figure 4 (under tachycardial conditions) and Figure S3 (under calm conditions). We did not observe any significant associations between the BMI z-score and the HRV parameters assessed in calm conditions, whereas significant non-linear associations were observed with HRV parameters assessed under tachycardial conditions.

#### Influence of Thinness

Spline regressions indicated a significant linear association between HRV and adiposity in the lowest ranges of adiposity (negative BMI z-scores). A BMI z-score <−2SD was associated with a lower HRV as measured by SDNN, RMSSD, LF, and HF. Compared to children with a normal BMI z-score (]−2DS to +1DS]), children with BMI z-score <−2SD had their RMSSD decreased by −30% (*p* = 0.03), their SDNN decreased by −21% (*p* = 0.03), their LF decreased by −37% (*p* = 0.04) and their HF decreased by −48% (*p* = 0.04). The LF/HF ratio was not significantly associated with adiposity (*p* = 0.25). Children with a BMI z-score ≤−2SD had also a higher heart rate [+6.8 (3.5) bpm; *p* = 0.05] (Table 4).

**Table 4:** Associations between HRV under tachycardial conditions and BMI z-scores, with and without adjustment for SBP (both sexes).

			**Not adjusted for SBP**		**Adjusted for SBP**			
			**BMI parameters**		**BMI parameters**		**SBP parameter**	
**HRV Parameter**	**BMI category**	***N***	**β (SE)**	***P*-value**	**β (SE)**	***P*-value**	**β (SE)**	***P*-value**
Heart Rate	Underweight	23	6.80 (3.50)	0.053	8.50 (3.46)	0.014	0.309 (0.074)	<0.0001
	Normal	213	ref		ref			
	Overweight	94	0.48 (1.64)	0.772	−1.06 (1.65)	0.52		
	Obese	73	−2.66 (2.24)	0.237	−5.38 (2.30)	0.02		
log(SDNN)	Underweight	23	−0.23 (0.11)	0.035	−0.24 (0.11)	0.029	−0.002 (0.002)	0.401
	Normal	213	ref		ref			
	Overweight	94	−0.02 (0.05)	0.668	−0.01 (0.05)	0.826		
	Obese	73	0.09 (0.07)	0.199	0.11 (0.07)	0.14		
log(RMSSD)	Underweight	23	−0.35 (0.16)	0.029	−0.39 (0.16)	0.013	−0.009 (0.003)	0.010
	Normal	213	ref		ref			
	Overweight	94	0.02 (0.07)	0.786	0.06 (0.08)	0.394		
	Obese	73	0.14 (0.10)	0.168	0.22 (0.11)	0.039		
log(LF)	Underweight	23	−0.47 (0.23)	0.045	−0.51 (0.23)	0.031	−0.007 (0.005)	0.147
	Normal	213	ref		ref			
	Overweight	94	−0.02 (0.11)	0.827	0.01 (0.11)	0.901		
	Obese	73	0.22 (0.15)	0.138	0.29 (0.16)	0.065		
log(HF)	Underweight	23	−0.65 (0.32)	0.044	−0.73 (0.32)	0.024	−0.015 (0.007)	0.033
	Normal	213	ref		ref			
	Overweight	94	0.02 (0.15)	0.9	0.09 (0.15)	0.542		
	Obese	73	0.27 (0.20)	0.187	0.4 (0.21)	0.06		
log(LF/HF)	Underweight	23	0.18 (0.15)	0.251	0.22 (0.16)	0.159	0.007 (0.003)	0.026
	Normal	213	ref		ref			
	Overweight	94	−0.04 (0.07)	0.557	−0.08 (0.07)	0.284		
	Obese	73	−0.05 (0.10)	0.623	−0.11 (0.10)	0.269		

*Linear regression models were adjusted for sex, age at measurement, maternal place of birth (French West Indies, other Caribbean Islands, Europe) and education (<5, 5–12, ≥12 years), TV time (<15 h or ≥15 h/week), videogames time (<4.5 h or ≥4.5 h/week), sport time (no sport, ≤ 2 h, >2 h/week) and obesogenic diet at the age of measurement*.

#### Influence of Fat Corpulence

No significant differences were observed in children with a moderately fat or fat corpulence when considering both sexes together.

However, after stratifying the analysis according to sex, we observed a global increase in all HRV parameters under tachycardial conditions in boys, particularly in the LF parameter in the 25 boys with a BMI z-score >2SD (β = 0.43 (0.18), *p* = 0.02). The associations in girls were all non-significant but tended to be in the opposite direction (Table 5).

**Table 5:** Associations between HRV (under tachycardial conditions) and BMI z-score, in boys and girls.

**HRV parameter**		**In boys**			**In girls**		
	**BMI category**	***N***	**β (SE)**	***P*-value**	***N***	**β (SE)**	***P*-value**
Heart rate	Underweight	9	9.56 (4.34)	0.029	3	−0.32 (7.34)	0.966
	Normal	147	ref		145	ref	
	Overweight	26	1.84 (2.68)	0.492	45	−0.41 (2.13)	0.849
	Obese	25	−3.31 (2.72)	0.225	9	−0.95 (4.21)	0.821
log(SDNN)	Underweight	9	−0.29 (0.13)	0.033	3	−0.07 (0.22)	0.758
	Normal	147	ref		145	ref	
	Overweight	26	0.00 (0.08)	0.964	45	−0.03 (0.06)	0.679
	Obese	25	0.13 (0.08)	0.138	9	−0.01 (0.12)	0.950
log(RMSSD)	Underweight	9	−0.45 (0.20)	0.024	3	0.02 (0.32)	0.957
	Normal	147	ref		145	ref	
	Overweight	26	−0.01 (0.12)	0.921	45	0.04 (0.09)	0.652
	Obese	25	0.24 (0.12)	0.055	9	−0.14 (0.19)	0.463
log(LF)	Underweight	9	−0.60 (0.29)	0.040	3	−0.33 (0.48)	0.492
	Normal	147	ref		145	ref	
	Overweight	26	0.03 (0.18)	0.868	45	−0.07 (0.14)	0.630
	Obese	25	0.43 (0.18)	0.020	9	−0.25 (0.27)	0.365
log(HF)	Underweight	9	−0.82 (0.40)	0.044	3	−0.03 (0.64)	0.961
	Normal	147	ref		145	ref	
	Overweight	26	−0.04 (0.25)	0.869	45	0.06 (0.19)	0.758
	Obese	25	0.48 (0.25)	0.063	9	−0.27 (0.37)	0.458
log(LF/HF)	Underweight	9	0.22 (0.20)	0.264	3	−0.30 (0.31)	0.336
	Normal	147	ref		145	ref	
	Overweight	26	0.07 (0.12)	0.560	45	−0.12 (0.09)	0.166
	Obese	25	−0.05 (0.12)	0.687	9	0.03 (0.18)	0.887

*Linear regression models were adjusted for age at measurement, maternal place of birth (French West Indies, other Caribbean Islands, Europe) and education (<5, 5–12, ≥12 years), TV time (<15 h or ≥15 h/week), videogames time (<4.5 h or ≥4.5 h/week), sport time (no sport, ≤ 2 h, >2 h/week) and obesogenic diet at the age of measurement*.

### Adjustment for SBP

After adjustment for SBP, the association of adiposity with HRV parameters was strengthened in the underweight children group (decreased HRV parameters and increased Heart rate) (Table 4). Significant or nearly significant associations were also observed in the obese children group: their mean heart rate was decreased by −5.4 (2.3) bpm (*p* = 0.02), their RMSSD was increased by 25% (*p* = 0.04), their LF by 34% (*p* = 0.06) and their HF by 49% (*p* = 0.06), compared to children with normal BMI.

### Association Between Cardiac Repolarization and Adiposity

We did not observe any significant association between the corrected ECG QTc interval length and the adiposity indicators, both at rest and under tachycardial conditions (Figure S4), in boys as in girls (data not shown).

## Discussion

This study, performed within a longitudinal mother-child cohort in a French West Indies general population, provides new information on early association between blood pressure and adiposity. Our study is one of the few to focus on the relationships between SBP, adiposity and HRV during the prebubertal period. In pre-pubertal period, we found that (i) Fat corpulence is already associated with higher blood pressure. (ii) This association is influenced by sex with higher blood pressure in the overweigh and obese boys. (iii) Abnormal corpulence (underweight or overweight) is already associated with abnormal autonomic control of heart rate as assessed by HRV analyses, but this was only significant under tachycardial conditions, suggesting an abnormal response to stress. A decrease in HRV is associated with BMI z-score ≤−2SD and an increase in LF in boys with BMI z-score >2SD. (iv) A significant association exists between higher pre-pubertal arterial pressure and the age at the adiposity rebound.

### SBP and Corpulence

In our study, the SBP was linearly associated with the adiposity indicators and the corpulence score. Our results suggest that the association between adiposity excess and SBP is already significant in pre-pubertal period. In adults the association between hypertension and overweight is well known [see Chiang et al. (24) for review (24)]. This relationship between SBP and corpulence has also been observed in a population of 198 white girls followed from 8 to 22 years by Guo et al. (5). An average increase of 1 kg/m^2^ in BMI resulted in an average increase of 1.2 mmHg in SBP and 0.6 mmHg in Diastolic Blood Pressure (5).

The childhood period has been further studied by Sun et al. in 2007 who analyzed serial data for 240 men and 253 women in the Fels Longitudinal Study to study the correlation between SBP in childhood and SBP in adulthood (30 years old). The authors identified a positive correlation between the BMI and the SBP in childhood data ranged from 0.26 to 0.48 for boys and from 0.20 to 0.56 for girls according to the categories of age; an increase in childhood BMI of 1 kg/m^2^ resulted in average increases in SBP of 1.0 ± 0.2 mmHg for boys and 0.8 ± 0.2 mmHg for girls (4). This work also showed that children with elevated SBP are at increased risk of hypertension later in life. The positive correlation between SBP and BMI observed in our study matches with the previous studies of Sun et al.

The study of Börnhorst et al. analyzed 3301 BMI trajectories of European children. The results described that the early adiposity rebound is a risk factor of metabolic syndrome at longer-term, particularly if this adiposity rebound appears between 9 months and 6 years. This study also showed that the BMI z-score was positively correlated in BP z-score [β = 0.19 (IC 95%, 0.05; 0.34)] (25). This correlation was however much less important than the one in our results.

Some authors explored the relationship between SBP and the other adiposity indicators. Recently, Dong et al. concluded that BMI could be a better predictor of elevated blood pressure than other adiposity indicators in children such as WtHR, skinfold thickness, or waist circumference (26). However, other authors, as Wicklow et al. found that waist circumference was a better predictor of SBP than BMI or waist circumference expressed relative to height or hip circumference (27). Our results contrast slightly with these ones. Indeed the SBP was positively associated with all adiposity indicators: z-BMI, fat mass, sum of skinfold thickness and WtHR and with no significant difference on association strength between them.

### HRV and Corpulence

In this study the significant associations between HRV and corpulence in the prepubertal period were observed during the 5 min tachycardial condition recorded before venipuncture without using a standardized stress test. Therefore, the period studied can only be considered as an approximation of a stressful condition. However, all the children were submitted to the same protocol, with tasks being proposed in the same order for all of them. Moreover, the significant associations observed during tachycardial condition always confirmed the trends observed in calm conditions (see Figure 4 and Figure S3). We think therefore that the significant linear association between HRV and adiposity in the lowest ranges of adiposity and the increase in LF observed in boys with fat corpulence during tachycardial condition could plausibly be interpreted as a significant abnormal autonomic control of heart rate revealed by stress.

In this study, a low BMI was associated with a tendency to a higher heart rate associated with a reduction in nearly all the HRV parameters studied suggesting a decrease of autonomic influence on heart rate control for these children. These results are close to the observation of Galetta et al. who observed a decrease of HRV in thin subjects (BMI < 20 kg/m^2^) compared to controls (BMI > 20 kg/m^2^) but an increase in HRV in a group of 25 patients with anorexia nervosa (range 13–20 years) (28). In the same way, Rechlin et al. analyzed a population of subjects from 16 to 40 years, enduring a period of anorexia nervosa. This study observed a decrease of the HRV in the spectral domain in Anorexia Nervosa when compared to control subjects (BMI > 18 kg/m^2^) (29).

These observations together with the observed changes in HRV and blood pressure variability induced by head-up tilt test in adults (30) argue for an autonomic insufficiency in underweighted children and in anorexia nervosa.

Our study covered much younger populations than the one described in these works. Thus, it is the only study that showed these results regarding the HRV and BMI relationship among the thinnest young children.

We have showed a significant association between fat corpulence and increase in LF which is known to be mainly determined by blood pressure ripples in boys during tachycardial condition. This was not observed in girls. This observation suggests that a sex dependent abnormal arterial pressure regulation is associated with BMI z-score >2SD in boys as early as in the pre-pubertal period. The previous published studies were not focused on the pre-pubertal period except the study by Michels et al that investigated 460 children (5–10 years) with Polar chest belts in supine position and found no association between BMI or fat mass with 5 min HRV parameters (31). In another case control-study including 36 older children [11.5 (0.8) years of age], in resting condition, obesity was associated with an increase in LFnu and LF/HF and with a decrease in HFnu but without significant differences between overweight and normal_weight children (32). This increase in LF/HF associated with a decrease in HF has also been reported in obese children studied between 8 and 16 years old (*n* = 16 vs. 40 controls) (33), at 9 ± 1.7 years (*n* = 91 in a cohort of 616) (34), at an average age of 11.5 years (*n* = 16 vs. 10 controls) (32) and at 13.9 ± 1.7 years (*n* = 15 vs. 12 controls) (8). Our results, together with these observations in older children, suggest a relative increase in sympathetic tone associated with fat corpulence, at least for some children. Thereby, it is plausible that a predisposition to adult hypertension could be identified before puberty in some overweighed and obese boys, opening a new window for precocious intervention or prevention to avoid or limit adult hypertension.

### QTc and Corpulence

In this study we did not find any association between the corrected QT length and the corpulence indicators. In adulthood, metabolic syndrome has been associated with QT lengthening (35) but our results are in agreement with the absence of correlation between obesity and length of the QT interval reported in a study concerning 4,655 <10 years Japanese children (36).

### Strengths and Limitations

The TIMOUN study is a large mother-child cohort, representative of the general French Caribbean population. The proportion of obese children observed in this study was in accordance with the known prevalence in Guadeloupe: 17.3% overweight children and 8.3 % children in situation of obesity in our study vs. 15.4 and 7.2 % respectively (12).

It is difficult to extrapolate our results to other populations because there is a lack of comparable studies at this age and including children from African ascendance. Future studies analyzing different populations seem necessary to better understand interrelations between SBP, HRV, and BMI.

The questionnaires administered to the parents were particularly detailed and we were able to adjust our results for many covariates such as sedentary lifestyle, sex, age, food habits, mother's level education, and maternal place of birth. All these methodological aspects together strengthen the observed results.

One strength of our study is our multidimensional approach of corpulence. We chose to describe the corpulence with the BMI z-score, but also with a composite corpulence score reflecting adiposity. Farah et al. showed in a population of obese normotensive adolescents (13–18 years) that central obesity, defined by waist circumference, was a better discriminator than general obesity, defined by BMI with regard to autonomic cardiac dysfunction (37). This adiposity indicator was validated in adulthood by the study of Janssen et al which showed, by using Magnetic Resonance Imaging, that BMI and Waist Circumference independently contributed to the prediction of non-abdominal, abdominal subcutaneous, and visceral fat in 341 adults (38). On the other hand a Canadian study by Khoury et al. analyzed in 14,493 subjects from 5 to 18 years, from 1999 to 2008, the ratio WtHR according to 3 categories (normal: <0.5; overweight: between 0.5 and 0.6; and obese: >0.6). It turned out that WtHR was highly correlated with the cardio-metabolic risk and the BMI (18). In our study, the corpulence score allowed a more complete definition of the adiposity and showed enhanced linkages with the BP, with regard to the results of the literature.

BP measurement in our study may suffer from limitations. In the Timoun protocol, BP was measured twice during a single-day medical examination. This may induce an overestimation of the children's BP, as suggested in previous studies (39). To limit this potential bias, we only considered the minimum value of SBP measured during the examination as an estimation of the everyday BP level. Moreover, our objective was not to identify or diagnose children with hypertension but to study BP as a continuous outcome to be compared across different categories of BMI and identify the shape of this association. As the overestimation is probably non-differential between children of different BMI categories, we can consider that the estimation of the association between SBP levels and BMI was not biased. Finally, the comparison of the mean SBP in our sample (mean age at examination: 92 months) with the 50th percentiles of the reference distributions (40) at 7 and 8 years for each sex indicated no significant higher values (For boys, 9.9 mmHg in the our sample vs. 9.7 mmHg at 7 years and 9.9 mmHg at 8 years in the reference population; For girls, 9.8 mmHg in our sample vs. 9.6 mmHg at 7 years and 9.8 mmHg at 8 years in the reference population).

According to our knowledge, our study is the first to identify a correlation between SBP and z-BMI in the pre-pubertal period but also to point out the interrelation between BMI and adiposity rebound and SBP. It is also the first to describe the influence of sex on the relation between arterial pressure and HRV suggesting the existence of alteration of cardiac autonomic regulation of arterial pressure associated with obesity in boys in the pre-pubertal period.

## Conclusion

The results of this study, together with the known associated risk of adult hypertension following the observation of high arterial blood pressure during childhood, argue for preventive intervention as early as in the infancy and pre-pubertal period, particularly in case of overweight or obesity, when the adiposity rebound is early, and in boys.

Thinness was associated with a reduction in nearly all the parameters of HRV studied, suggesting a decrease of autonomic influence control for these children. This could be involved in the cardio-vascular and metabolic alterations associated with low corpulence as in anorexia.

## Data Availability

The datasets generated for this study are available on request to the corresponding author.

## Ethics Statement

Participants provided written informed consent for participation, and the study was approved by the Guadeloupean Ethics Committee for studies involving human subjects.

## Author Contributions

SC, PP, FR, and LMu contributed conception and design of the study. FR organized medical examinations. CM and LMi set up data collection and management. MG, MD, M-BS, and PP performed signal analysis. NC proposed and performed statistical analyses. MG, NC, MD, FR, and PP wrote the first draft of the manuscript. All authors contributed to manuscript revision and read and approved the submitted version.

### Conflict of Interest Statement

The authors declare that the research was conducted in the absence of any commercial or financial relationships that could be construed as a potential conflict of interest.

## Footnotes

^1^Doyen M, Ge D, Beuchée A, Carrault G, Hernandez A. Robust, real-time QRS detector based on a multi-feature probabilistic method. Submitted for publication.^**^ https://hal.archives-ouvertes.fr/hal-01619803
