# Supplementary material for: Blood Pressure, Heart Rate Variability, and Adiposity in Caribbean Pre-pubertal Children
Source: Front Pediatr. 2019 Jul 10;7:269. doi: 10.3389/fped.2019.00269 (PMC6635797; doi:10.3389/fped.2019.00269)
Supplement: Supplementary file 1 [file Data_Sheet_1.docx]

**SUPPLEMENTAL MATERIAL**

**Blood Pressure, Heart Rate Variability, and Adiposity in Caribbean Pre-pubertal Children**

Morgane Grandemange, Nathalie Costet, Matthieu Doyen, Christine Monfort, Léah Michineau, Marie-Béatrice Saade, Luc Multigner, Sylvaine Cordier, Patrick Pladys and Florence Rouget

**Computation of BMI z-scores**

The tabulated fitted values of Box-Cox power, median and coefficient of variation corresponding to height t are denoted by L(t), M(t) and S(t), respectively.

y = BMI; t = age

Calculate :

Compute the final z-score (Z^*^_ind_ ) of the child for that indicator as:

where

SD3*pos* is the cut-off 3 SD calculated at t by the LMS method:

SD3*neg* is the cut-off -3 SD calculated at t by the LMS method:

SD23*pos* is the difference between the cut-offs 3 SD and 2 SD calculated at t by the LMS method :

and SD23*neg*  is the difference between the cut-offs -2 SD and -3 SD calculated at t by the LMS method:

**Figure S1: Protocol of the medical examination of the children of the Timoun cohort at age 7 (times at SBP and HRV measurements)**

**
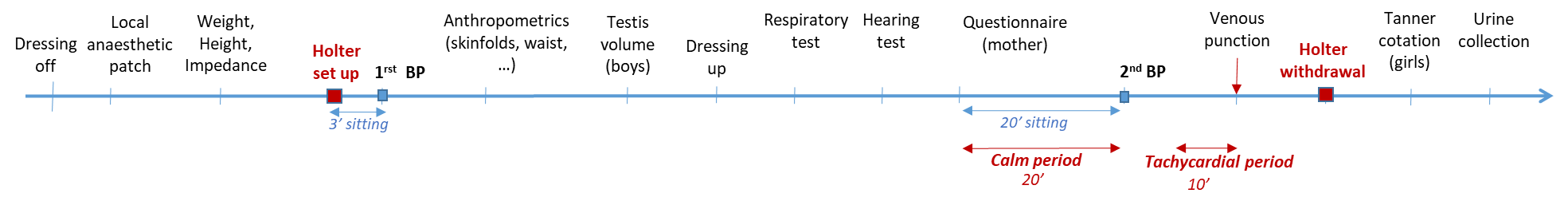
**

**Figure S2: First factorial plan from the Principal Component Analysis of the 4 adiposity indicators to define the “adiposity score”.**

**
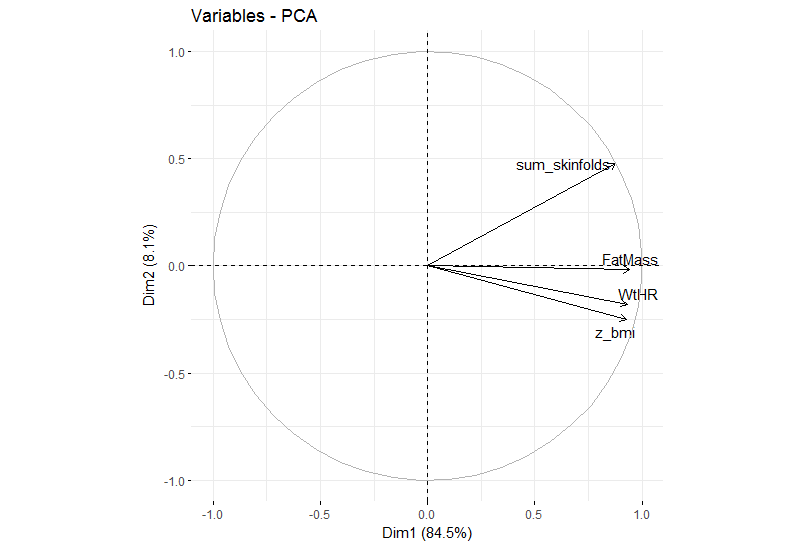
**

**Figure S3:**

**Association between HRV parameters and BMI z-score.** Restricted cubic splines regression models, adjusted for age at measurement, maternal place of birth and education, TV and videogames times, sport time, indicator of obesogenic food consumption.


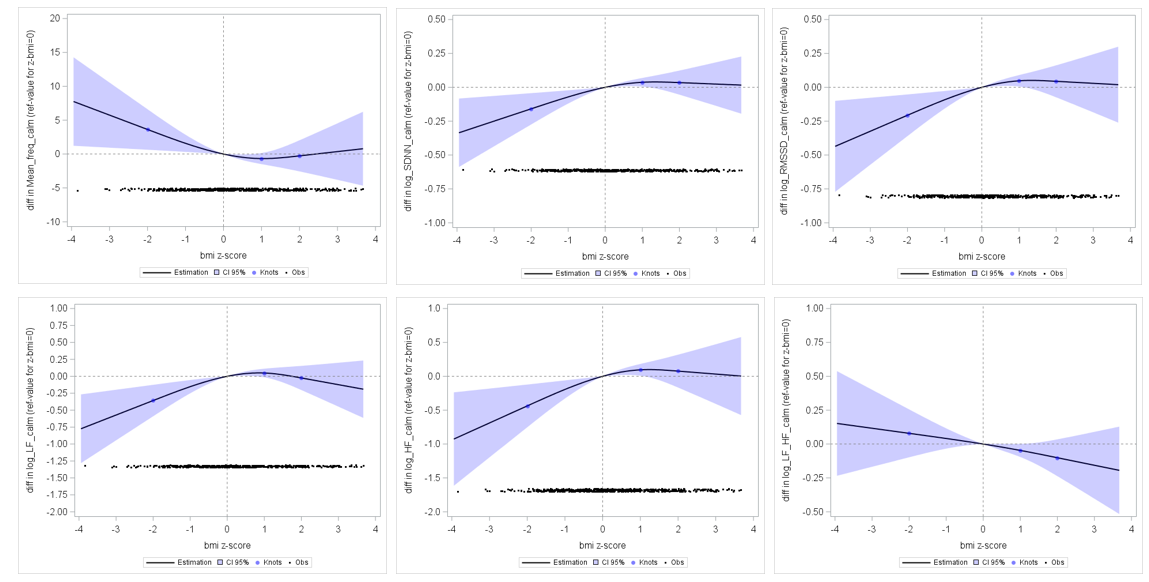


Interpretation:

The reference value for the BMI z-score (indicated with a vertical dotted line) was 0.

The y-axis represents the mean variation in HRV parameters associated to each BMI z-score level, compared to the mean level of HRV parameters associated with a 0 BMI z-score.

The black points in the bottom of the graphs represent the number of children observed in the study at each level of the BMI z-score.

The blue points on the spline regression curve represent the knots, placed at the WHO thresholds defining underweight and overweight and obesity (respectively -2SD, +1SD, +2 SD).

The bluish surface around the estimated regression curve represents the 95% confidence interval of the values of variation in HRV parameters estimated from the spline regression model.

**Figure S4: Association between corrected ECG QT interval (ms) (calm conditions) and adiposity indicators (both sexes).**


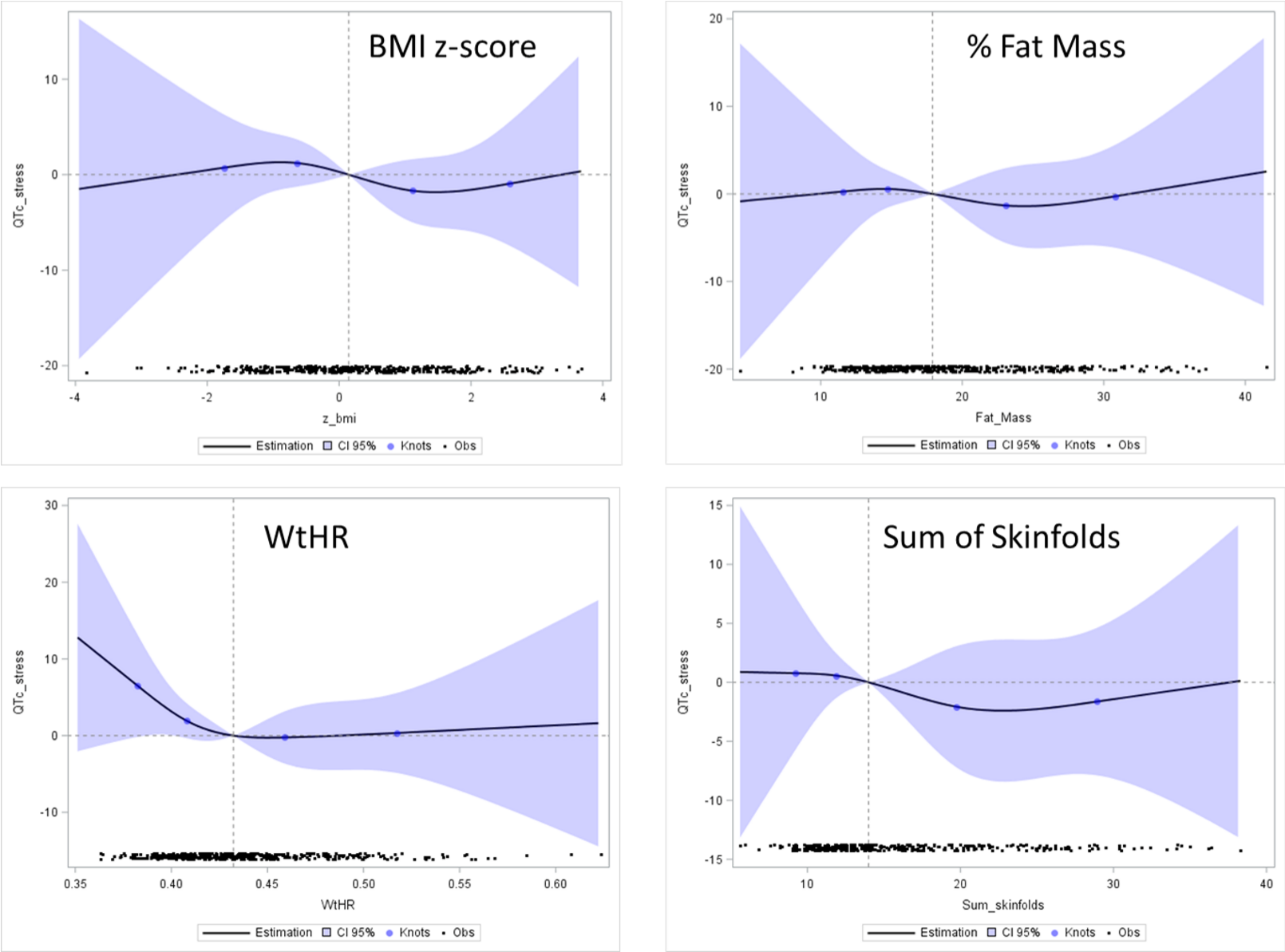


Restricted cubic splines regression models, adjusted for sex of the child, age at measurement, maternal place of birth and education, TV and videogames time, sport time, indicator of obesogenic food consumption.

Interpretation:

The reference value for the adiposity indicator (indicated with a vertical dotted line) was 0 for the z_bmi and the median level for the other indicators.

The y-axis represents the mean variation in corrected QT interval associated to each adiposity level, compared to the mean level of corrected QT interval associated with the median level of the adiposity indicator.

The black points in the bottom of the graphs represent the number of children observed in the study at each level of the adiposity indicator.

The blue points on the spline regression curve represent the knots, set at the 5 ^th^, 25^th^, 75^th^, and 95^th^ percentiles of the adiposity indicator.

The bluish surface around the estimated regression curve represents the 95% confidence interval of the values of variation in corrected QT interval estimated from the spline regression model.

| **Table S1. Adiposity and cardiac characteristics of the children included in the SBP analysis (N=575)** | | | | | |
| --- | --- | --- | --- | --- | --- |
|  |  | | | | |
|  | **Boys (N=283)** | |  | **Girls (N=292)** | |
| **Characteristics** | N | % or mean(std)[min-max] or median(IQR) [min-max] |  | N | % or mean(std)[min-max] or median(IQR) [min-max] |
|  |  |  |  |  |  |
| **Adiposity measurements** |  |  |  |  |  |
| **Weight (kg)** | 282 | 28.3 (6.1) [15.6; 52.7] |  | 291 | 27.9 (5.8) [16.6; 53.3] |
| **Height (cm)** | 283 | 130.5 (5.9) [115.5-146] |  | 292 |  |
| **BMI (kg/m²)** | 282 | 16.5 (2.7) [11.6; 25.5] |  | 291 | 16.4 (2.5) [11.7; 27.3] |
| **BMI z-score** | 282 | 0.2 (1.5) [-3.9; 3.7] |  | 291 | 0.2 (1.2) [-3.1; 3.5] |
| **BMI categories** |  |  |  |  |  |
| Underweight, ≤ -2SD | 12 | 4.3 |  | 5 | 1.7 |
| Normal, ]-2SD; +1SD] | 195 | 69.1 |  | 211 | 72.5 |
| Overweight, ]+1SD; +2SD] | 33 | 11.7 |  | 54 | 18.6 |
| Obese, > 2SD | 42 | 14.9 |  | 21 | 7.2 |
| **% Fat Mass** | 276 | 18.2 (5.8) [4.3; 37.2] |  | 289 | 20.4 (6.5) [8.0; 41.5] |
| **Sum of triceps + subscapular skinfolds (mm)** | 240 | 15.0 (6.4) [5.0; 43.7] |  | 255 | 17.9 (6.9) [5.9; 48.3] |
| **Waist-to-height ratio** | 283 | 0.44 (0.04) [0.35; 0.62] |  | 288 | 0.44 (0.05) [0.36; 0.59] |
| **Cardiac measurements** |  |  |  |  |  |
| SBP (mmHg) | 282 | 99 (10) [79-133] |  | 292 | 98 (13) [70-136] |

| **Table S2. Associations between HRV under calm conditions and BMI z-score, with and without adjustment for SBP (both sexes)** | | | | | | | | | | |
| --- | --- | --- | --- | --- | --- | --- | --- | --- | --- | --- |
|  |  |  |  | **No adjustment for SBP** | |  | **Adjustment for SBP** | | | |
| **HRV** |  |  |  | **Parameters for BMI** | |  | **Parameters for BMI** | | **Parameter for SBP** | |
| **parameter** | **BMI category** | **N** |  | **β (SE)** | **P-value** | | **β (SE)** | **P-value** | **β (SE)** | **P-value** |
|  |  |  |  |  |  |  |  |  |  |  |
| **Heart Rate** | Underweight | 23 |  | 2.85 (3.11) | 0.36 |  | 3.5 (3.13) | 0.264 | 0.121 (0.067) | 0.073 |
|  | Normal | 213 |  | ref |  |  | ref |  |  |  |
|  | Overweight | 94 |  | 1.04 (1.45) | 0.473 |  | 0.43 (1.49) | 0.771 |  |  |
|  | Obese | 73 |  | -1.57 (1.99) | 0.43 |  | -2.65 (2.08) | 0.203 |  |  |
|  |  |  |  |  |  |  |  |  |  |  |
| **log(SDNN)** | Underweight | 23 |  | -0.18 (0.12) | 0.128 |  | -0.19 (0.12) | 0.109 | -0.002 (0.003) | 0.374 |
|  | Normal | 213 |  | ref |  |  | ref |  |  |  |
|  | Overweight | 94 |  | -0.02 (0.06) | 0.665 |  | -0.01 (0.06) | 0.838 |  |  |
|  | Obese | 73 |  | 0.09 (0.08) | 0.229 |  | 0.11 (0.08) | 0.154 |  |  |
|  |  |  |  |  |  |  |  |  |  |  |
| **log(RMSSD)** | Underweight | 23 |  | -0.19 (0.16) | 0.233 |  | -0.21 (0.16) | 0.194 | -0.004 (0.003) | 0.282 |
|  | Normal | 213 |  | ref |  |  | ref |  |  |  |
|  | Overweight | 94 |  | -0.02 (0.07) | 0.751 |  | 0 (0.08) | 0.956 |  |  |
|  | Obese | 73 |  | 0.11 (0.1) | 0.255 |  | 0.15 (0.11) | 0.158 |  |  |
|  |  |  |  |  |  |  |  |  |  |  |
| **log(LF)** | Underweight | 23 |  | -0.35 (0.23) | 0.13 |  | -0.36 (0.23) | 0.119 | -0.003 (0.005) | 0.499 |
|  | Normal | 213 |  | ref |  |  | ref |  |  |  |
|  | Overweight | 94 |  | -0.11 (0.11) | 0.303 |  | -0.09 (0.11) | 0.412 |  |  |
|  | Obese | 73 |  | 0.08 (0.15) | 0.582 |  | 0.12 (0.15) | 0.447 |  |  |
|  |  |  |  |  |  |  |  |  |  |  |
| **log(HF)** | Underweight | 23 |  | -0.43 (0.31) | 0.17 |  | -0.47 (0.32) | 0.142 | -0.007 (0.007) | 0.305 |
|  | Normal | 213 |  | ref |  |  | ref |  |  |  |
|  | Overweight | 94 |  | 0.01 (0.15) | 0.948 |  | 0.05 (0.15) | 0.756 |  |  |
|  | Obese | 73 |  | 0.19 (0.2) | 0.335 |  | 0.26 (0.21) | 0.217 |  |  |
|  |  |  |  |  |  |  |  |  |  |  |
| **log(LF/HF)** | Underweight | 23 |  | 0.08 (0.18) | 0.647 |  | 0.1 (0.18) | 0.567 | 0.004 (0.004) | 0.356 |
|  | Normal | 213 |  | ref |  |  | ref |  |  |  |
|  | Overweight | 94 |  | -0.12 (0.08) | 0.154 |  | -0.14 (0.09) | 0.114 |  |  |
|  | Obese | 73 |  | -0.11 (0.12) | 0.331 |  | -0.14 (0.12) | 0.238 |  |  |

Linear regression models were adjusted for sex, age at measurement, maternal place of birth (French West Indies, other Caribbean Islands, Europe) and education (<5, 5-12, ≥12 yrs), TV time (<15h or ≥15h / week), videogames time (<4.5h or ≥4.5h / week), sport time (no sport, ≤ 2 hours, > 2 hours/week) and obesogenic diet at the age of measurement.
